# BAG2 Promotes Proliferation and Metastasis of Gastric Cancer via ERK1/2 Signaling and Partially Regulated by miR186

**DOI:** 10.3389/fonc.2020.00031

**Published:** 2020-01-31

**Authors:** Lisha Sun, Guanglei Chen, Anqi Sun, Zheng Wang, Haibo Huang, Ziming Gao, Weitian Liang, Caigang Liu, Kai Li

**Affiliations:** ^1^Department of Surgical Oncology, The First Hospital of China Medical University, Shenyang, China; ^2^Department of Breast Surgery, Shengjing Hospital of China Medical University, Shenyang, China; ^3^Department of Otorhinolaryngology, The First Hospital of China Medical University, Shenyang, China

**Keywords:** gastric cancer, BAG2, miR186, iTRAQ proteomics technology, therapeutic target

## Abstract

Bcl2-associated athanogene (BAG)2 as a co-chaperone has been demonstrated to be involved in tumor growth and metastasis, but its biological function in gastric cancer remains unknown. Here, we reported that BAG2 was highly expressed in gastric cancer cell lines and tissues, indicating poor prognosis. High expression of BAG2 was significantly associated with T stage and differentiation level of gastric cancer (*P* < 0.001). Functional experiments revealed that *BAG2* knockdown in gastric cancer cells inhibited the proliferation, invasion and migration of cells through AKT/mTOR and extracellular regulated kinase (ERK) pathways. Proteomic analysis identified that BAG2 may be involved in the regulation of mitogen-activated protein kinase (MAPK) pathway. In addition, immunoprecipitation showed that BAG2 could bind to ERK1/2. Luciferase reporter assay and Western blot verified that *BAG2* was down-regulated by miR186. Taken together, our findings may reveal the basic function of BAG2 and uncover a potential therapeutic target for gastric cancer.

## Introduction

Gastric cancer is a highly aggressive malignancy that is currently the third cause of cancer death (1). Although the overall survival (OS) rate of gastric cancer has improved in recent years, there are still many patients with recurrence and metastasis (2). To improve the prognosis of gastric cancer patients, it is important to identify predictive biomarkers and potential therapeutic targets to develop more effective treatment strategies. BAG2 (Bcl-2-associated athanogene 2) is a protein identified by two-hybrid with heat shock protein 70 (Hsp70) as bait (3, 4), which contains at least three domains, BAG domain, BNB (Brand New Bag) domain, and amino terminal domain (5). Functioning as a co-chaperone, BAG2 interacts with the carboxyterminal region of Hsp70/C-terminal interacting protein, and then regulates the biological activity of molecular chaperones, including ubiquitin proteasome system (3, 6–8).

The roles of BAG2 in cancer are not well-studied. It has been reported that high expression of BAG2 can induce p21-dependent aging and subsequent carcinogenic stagnation (9). It also has been shown that BAG2 can promote apoptosis in thyroid cancer in response to proteasome inhibitors (10). However, in recent years, further studies have shown that *BAG2* plays a pivotal role as an oncogene. BAG2 is reported as highly expressed in tumors, including colorectal cancer, breast cancer, and head and neck squamous cell carcinoma (11). BAG2 was also highly expressed in triple negative breast cancer and associated with cancer metastasis (12). Currently, the role of BAG2 in gastric cancer remains elusive.

## Materials and Methods

### Tissue Samples and Cell Culture

Gastric tissue samples were obtained from the patients in the First Hospital of China Medical University. Human gastric cancer cell line MKN45 were accessed from American Type Culture Collection (ATCC, USA). GES-1, MGC803, SGC7901, and HGC27 cell lines were purchased from the Cell Bank of typical Culture Preservation Committee of Chinese Academy of Sciences (China). Except that MGC803 cells were cultured in high glucose DMEM medium containing 10% FBS (Sciencell), the other cell lines were cultured in RPMI 1640 medium supplemented with 10% FBS (Sciencell) in a humidified atmosphere of 5% CO_2_ at 37°C.

### *BAG2*-Specific siRNAs and miR186 Mimics Transfection

*BAG2*-specific siRNAs and miR186 mimics were transfected into the HGC27 cell line using X-tremeGENE siRNA Transfection Reagent (Roche). Briefly, HGC27 cells were cultured in 6-well plates overnight and when the cells reached 70% of confluency, the cells were transfected with miR186 mimics, *BAG2*-specific siRNAs (1# *siBAG2*, 5′-GGGAAGAACUCUCACCGUUTT-3′; 2# *siBAG2*, 5′-GGGAAAUGCCAAGAGUCAUTT-3′; 3# *siBAG2*, 5′-GCUGAAAGCAGAUUCAAUUTT-3′) or control siRNA (5′-UUCUCCGAACGUGUCACGUTT -3′).

### *BAG2*-shRNAs Transfection

HGC27 cells (5 × 10^5^ cells/well) were cultured overnight and transduced with lentivirus for expressing control shRNA (5′-TTCTCCGAACGTGTCACGT-3′) or *BAG2*-specific shRNA (5′-GATCAGAAGTTTCAATCCATA-3′). The cells were treated with 5 μg/ml of puromycin to generate stably *BAG2* knockdown HGC27/*shBAG2* cells or negative control HGC27/NC cells. The efficacy of *BAG2* knockdown was verified by Western blot.

### Western Blot

The different groups of cells were lyzed in RIPA lysis buffer containing PMSF, Protease/Phosphatase Inhibitor Cocktail which came from Cell Signaling Technology (CST, 5872). After being centrifuged, the concentrations of total proteins were determined. Individual cell lysate samples (20 μg/lane) were separated by SDS-PAGE on 6-12% gels and transferred onto polyvinylidene difluoride (PVDF) membranes (Millipore). The membranes were blocked with 5% BSA in TBST and incubated overnight at 4°C with primary antibodies. The antibodies included human BAG2 (Thermofisher, PA5-30922), AKT (4691), mTOR (2972), ERK1/2 (4695), p38 (9212), and GAPDH (5174) from CST; E-cadherin(ab15148), N-cadherin (ab18203), MMP9 (ab38898), and Snail (ab180714) from Abcam; Vimentin (wl00742) from Wanleibio (China). The bound antibodies were detected with horseradish peroxidase (HRP)-conjugated secondary antibodies (at 1:10000 dilution, CST, 7074). The immunoblotting signals were visualized using the enhanced chemiluminescent reagents. The relative levels of individual target proteins to the control GAPDH were determined by the densitometric analysis using the ImageJ software.

### Immunohistochemistry (IHC)

Paraffin-embedded tissue sections from gastric cancer patients were used for immunohistochemistry of BAG2. After dewaxing and hydration, sections were incubated with 3% H_2_O_2_ to block endogenous enzymes before incubation with primary antibody of BAG2 (Thermofisher, PA5-72897, dilution of 1:1000) at 4°C overnight. the sections were incubated with the secondary antibody (MaxVision HRP-polymer anti-mouse/rabbit IHC Kit, 5002, Maixin). The immunostaining was visualized using diaminobenzidine. At each step, sections were rinsed several times with PBS. BAG2 expression was classified semi-quantitatively as follows: 0, no staining; 1, partial staining; 2, mild to moderate circumferential staining; and 3, strong staining. A score of 0 or 1 was considered low expression of BAG2. A score of 2 or 3 was considered high expression of BAG2.

### Cell Counting Kit 8 (CCK8) Assay

The cell viability was determined by CCK8 assay (Dojindo, Japan). Cells were transfected with *BAG2*-siRNA or negative control siRNA in 6-well plates for 24 h. Then these cells were transferred into 96-well plates and incubated for different time periods. Then, new medium with 10% CCK8 solution was added into each well and incubated for 2 h. The cell viability was analyzed at 450 nm.

### Transwell Assay

The impact of *BAG2* knockdown on the invasion and migration of gastric cancer cells was determined by transwell assay. For invasion assay, membranes of the top chambers were coated with Matrigel (BD) and pre-hydrated in serum-free medium (13). Briefly, control or HGC27/*siBAG2* cells (2 × 10^4^ cells/well) were loaded on the upper chamber of 24-well transwell plates (8-μm pore size, Corning) in FBS-free medium. The bottom chambers were filled with complete medium. After 24h, cells on the upper surface of the membrane were removed using cotton swabs and migrated cells on the bottom surface were stained with 0.1% crystal violet. The numbers of migrated cells in five randomly selected fields were counted under a phase contrast microscope in a blinded manner.

### EdU Cell Proliferation Assay

Cell proliferation was determined using the kFluor555-EdU cell proliferation kit (Keygentec, China). Cells were cultured in 6-well plates at 2 × 10^4^ cells/well overnight and incubated with EdU (10 μM) for 2 h. Followed washing cells with PBS, cells were incubated with 4% paraformaldehyde for fixation. Then 2 mg/mL glycine was added for 5 min, washed with 3% BSA in PBS, then added 0.5% tritonX-100 for 20 min. Click-iT reaction mixture were prepared following the manufacturer's protocol. Samples were observed with a fluorescence microscope immediately after DNA was stained with Hoechst33342.

### Annexin V-FITC/PI Cell Apoptosis Assay

Cells were digested with trypsin and washed with PBS. After adjusting the cell concentration to 2 × 10^5^ cell/tube, cells were resuspended in 195 μL of binding buffer and each sample was added with 5 μL of Annexin V-FITC (BD), incubated at room temperature for 15 min in the dark. Followed washing with binding buffer, propidium iodide (PI) was added in each sample and incubated for 5 min. Then, samples were analyzed by flow cytometry.

### Colony Formation Assay

HGC27/NC and HGC/*shBAG2* cells were cultured in 6-well plates at a density of 2,000 cells/well overnight. Subsequently, cells were washed with PBS and supplied with fresh medium with serum every 3 days. After 12 days, the colonies were stained using 0.1% crystal violet, and photographed under a microscope (Nikon, Japan). The experiment was performed in triplicate.

### Quantitative RT-PCR

The expression of miR186 and *BAG2* mRNA in gastric cancer cell lines were performed by a real-time PCR system (PrimeScript™ RT reagent Kit, Takara). Briefly, total RNA was isolated from HGC27 cells using TRIzol reagent (Invitrogen, USA) according to the manufacture's instruction. The primers were synthesized by Taihegene (China). Expression of miR186 was normalized to *U6* (endogenous control for miRNA) and *BAG2* mRNA was normalized to Actin (endogenous control), and data was processed by the 2^−(ΔΔ*Ct*)^ method.

### Luciferase Reporter Assay

HGC27 cells were prepared in 24-well plates at a density of 5 × 10^4^ cells/well overnight before transfection. Plasmid pmirGLO (Genechem, China) containing the *BAG2* wild type (WT) and three kinds of *BAG2* mutant type (Mut1, Mut2, and Mut1+2) were employed in the luciferase reporter assay. MiR186 mimics were transfected into the cells using X-tremeGENE HP DNA Transfection Reagent (Roche). After incubated for 48 h, luciferase activity was measured by a Dual-luciferase Reporter Assay System (Promega, USA) according to manufacturer's protocol.

### Immunoprecipitation

HGC27 cells were harvested and lysed in cold RIPA lysis buffer containing protease inhibitors, followed by centrifuging. The cell lysates (50 μg/tube) were incubated with anti-BAG2, anti-ERK1/2 or control isotype IgG (2 μg) with gentle agitation at 4°C overnight. Subsequently, the reactive mixtures in individual tubes were added with 70 μl of protein G with TBST and the bound proteins were eluted with 2 × SDS loading buffer. The eluted proteins were subjected to SDS-PAGE and immunoblotting with anti-BAG2 and anti-ERK1/2, respectively.

### Isobaric Tags for Relative and Absolute Quantitation (iTRAQ) Technology

The protein was extracted from HGC27/NC and HGC27/*shBAG2* cells. After the concentration is determined by BCA kit, the protein was processed of disulfide bond cleavage and reductive alkylation of proteins with DTT and iodoacetamide, and then the protein was hydrolyzed by trypsin. Labeled with iTRAQ labeling reagent, the peptide was mixed in the same amount, and the high PH reverse pre-separation was carried out. The pre-separated components were analyzed by low PH nano-HPLC-MS/MS (Orbitrap Fusion), and the data acquisition mode was data-dependent acquisition mode. The proteins were analyzed by GO (Gene Ontology) and KEGG, and the differentially expressed proteins (DEPs) between groups was analyzed at the same time.

### Statistical Analysis

Data are expressed as the mean ± SD. The difference among the groups was analyzed by one-way ANOVA and the difference between two groups was analyzed by Student's *T*-test. The χ^2^ test or linear by linear association was used to assess the correlation between BAG2 expression and clinicopathological features. The disease-free survival (DFS) of each group of patients was estimated by the Kaplan-Meier method and analyzed by Log-rank test. All statistical analyses were performed using the SPSS version 23.0 software. A *P* < 0.05 was considered statistically significant.

## Results

### High Expression of *BAG2* in Gastric Carcinoma Results in Poor Prognosis

**Figure 1:**
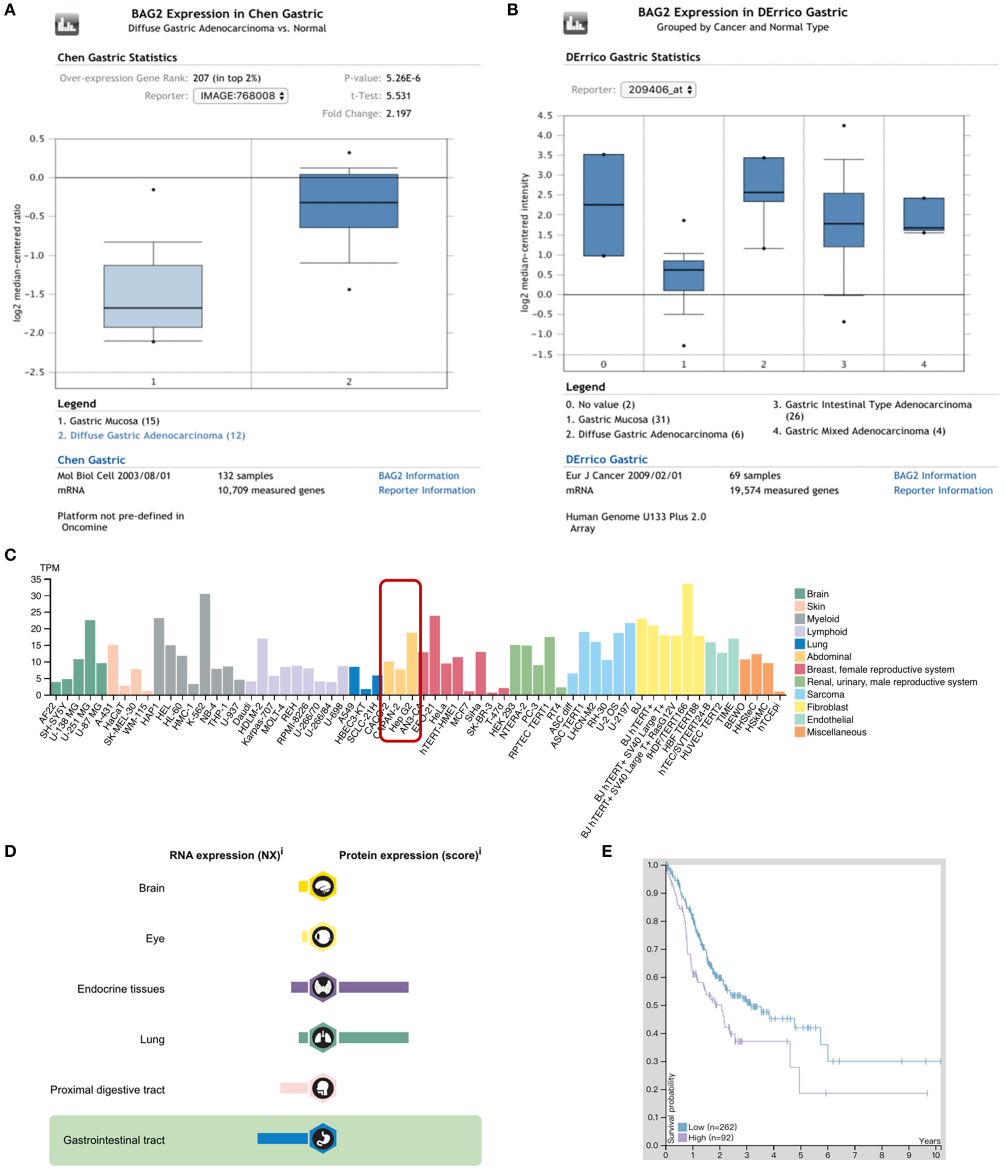
Expression of *BAG2* in gastric carcinoma in Oncomine and Human ProteinAtlas databases. **(A)** Expression of *BAG2* in gastric carcinoma is higher than normal gastric mucosa (Oncomine); **(B)** Expression of *BAG2* in different types of gastric carcinoma (Oncomine); **(C)** *BAG2* is highly expressed in various abnormal tumor cell lines (Human Protein Atlas); **(D)** Distribution and proportion of *BAG2* mRNA and protein expression in various tissues of human body, including gastrointestinal tract (Human Protein Atlas); **(E)** Prognostic analysis of *BAG2* in gastric cancer showed that the high expression of *BAG2* suggested a poor prognosis (The purple line represents high expression of *BAG2*, and the blue line represents low expression of *BAG2*. When the FPKM cutoff value of *BAG2* is 4.6, the survival time has significant statistical significance (Human Protein Atlas).

Oncomine database was used to query the differentially expressed genes (DEGs) between cancer tissues and para-cancerous gastric mucosa of patients with gastric cancer. We found that the expression of *BAG2* in gastric cancer was significantly higher than that in normal gastric mucosa (*P* < 0.01; Figure 1A), and the expression of *BAG2* in diffuse gastric cancer was higher than that in intestinal adenocarcinoma (Figure 1B). Meanwhile, we analyzed *BAG2* in immortal cell lines, tissues, and tumor pathology in Human Protein Atlas database. It was revealed that *BAG2* was highly expressed in abdominal tumor cell lines, including CACO-2, CAPAN-2, and Hep G2 (Figure 1C). However, in normal gastrointestinal tissues, *BAG2* mRNA was at a low level and BAG2 protein was not often detected (Figure 1D). As for tumor pathology, the prognosis of *BAG2* was analyzed by RNA samples from 354 cases of gastric cancer in The Cancer Genome Atlas. It was disclosed that the high expression of *BAG2* in gastric cancer is associated with poor prognosis (the cutoff value of FPKM was 4.6, Log-rank *P* = 0.006) (Figure 1E).

### *BAG2* Knockdown Inhibits Proliferation, Promotes Apoptosis, and Inhibits Invasion and Migration of Gastric Cancer Cells

**Figure 2:**
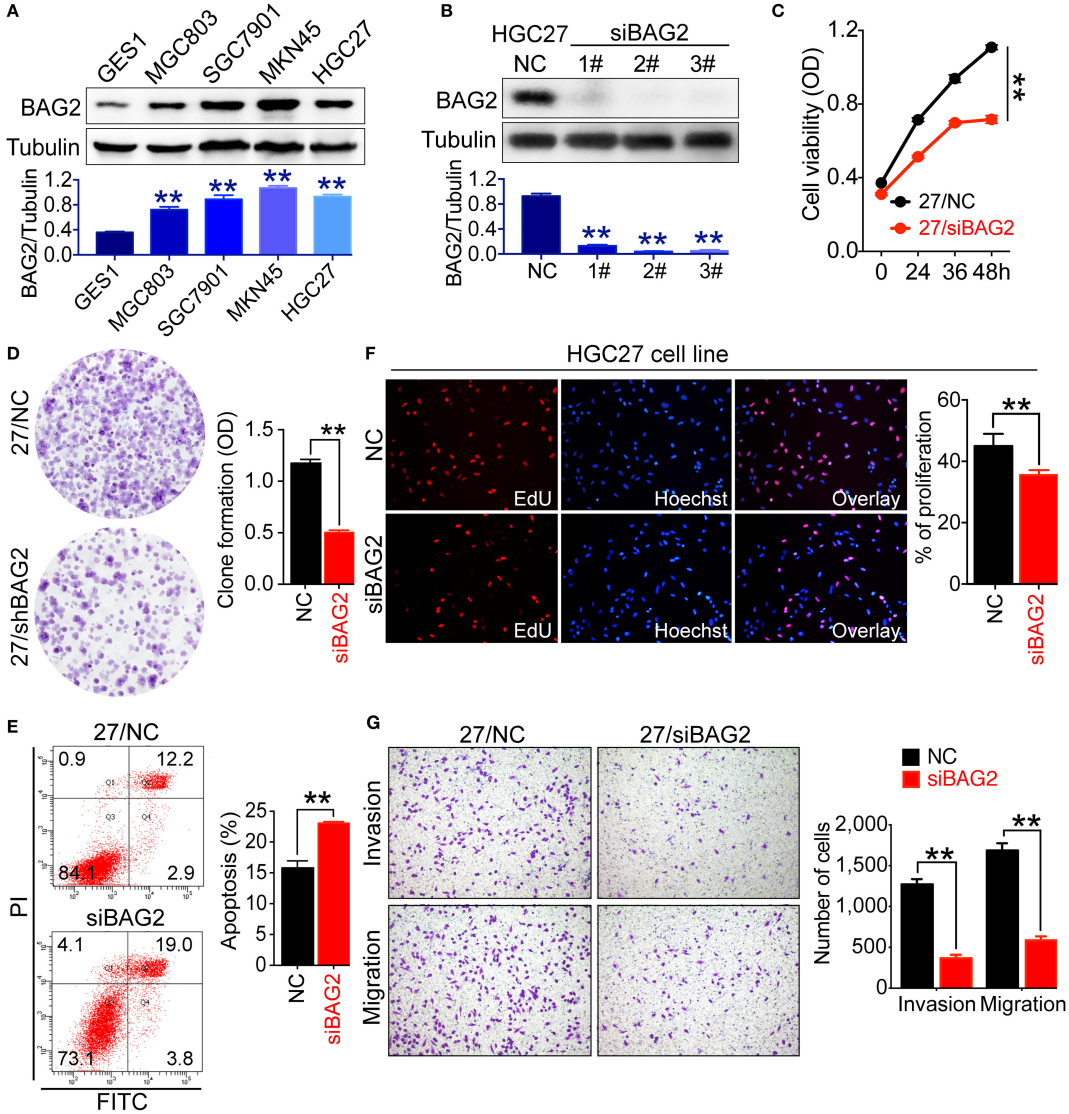
Biological function of BAG2 in gastric cancer cells. **(A)** Expression of BAG2 in GES1 and several gastric cancer cell lines (MGC803, SGC7901, MKN45, HGC27) showed that BAG2 expressed lowest in GES1 cell line; **(B)** Interference efficiency of *BAG2*-specific siRNAs; **(C–G)** Effect of *BAG2* knockdown on cell viability **(C)**, colony formation and cell apoptosis **(D)**, cell proliferation **(F)**, invasion and migration **(G)** of gastric cancer cells. **P < 0.01. 27/NC stands for HGC27/NC and 27/*siBAG2* stands for HGC27/*siBAG2*.

To verify the above-mentioned results, we tested the expression of BAG2 in normal gastric mucosa cell line GES1 and gastric cancer cell lines with different differentiations, such as HGC27 (undifferentiated), MKN45 (poorly differentiated), MGC803 (poorly differentiated), and SGC7901 (moderately differentiated). Results showed that the expression of BAG2 in GES1 was significantly lower than that in various gastric cancer cell lines (Figure 2A). Next, we used the *BAG2*-specific siRNA to knock down *BAG2* expression in HGC27 cells, and *BAG2* knockdown on the biological behavior of gastric cancer cells was observed. Firstly, we assessed the knockdown efficiency of three different *BAG2*-siRNA, and observed the knockdown efficiency of all three siRNAs was more than 90%, we then randomly selected *BAG2*-siRNA (2#) to carry out the follow-up functional experiments (Figure 2B).

Then we analyzed cell viability and proliferation of *BAG2* knockdown gastric cancer cells using Cell Counting Kit-8 (CCK8) and colony formation assay, respectively. Knockdown of *BAG2* significantly inhibited the cell viability and proliferation of HGC27 cells (Figures 2C,D). Using EdU reagent and Annexin V-FITC/PI cell apoptosis kit, we found that the proliferation ability of gastric cancer cells was markedly inhibited, and the apoptotic cells were significantly increased in *BAG2* knockdown cells (Figures 2E,F). In addition, we also examined the influences of BAG2 on the invasion and migration of gastric cancer cells by Transwell chamber. The results showed that knockdown of BAG2 could notably inhibit the invasion and migration of gastric cancer cells (Figure 2G).

### BAG2-Related Differential Expressed Proteins by iTRAQ Technology

**Figure 3:**
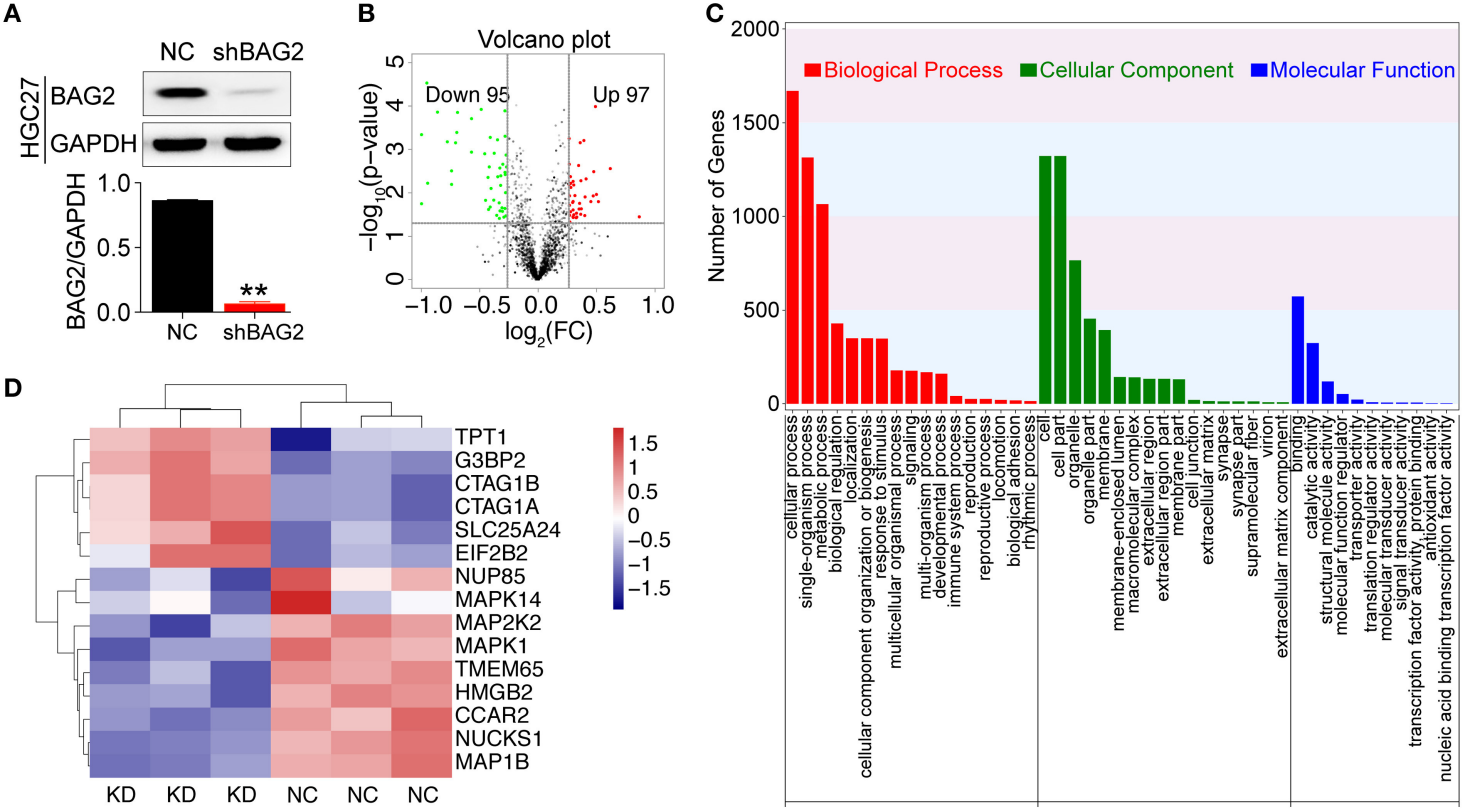
Proteomic analysis after knockdown *BAG2*. **(A)** Efficiency verification of shRNA for *BAG2* knockdown in the HGC27 cell line (** *P* < 0.01); **(B)** Volcanic map of DEPs showed 97 up-regulated and 95 down-regulated (when the difference multiple was more than 1.2 times or <1/1.2 times. The *p*-value was <0.05 by *t*-test, which was regarded as DEPs); **(C)** iTRAQ proteomic analysis of biological processes (BP), cell component (CC) and molecular function (MF) in which BAG2 may be involved; **(D)** Clustering map of partial BAG2-related DEPs with statistical differences.

Followed by transduction with lentiviral shRNA for *BAG2* to stably reduce its expression within the 10th generation (Figure 3A), we utilized iTRAQ technology for further bioinformatics analysis. It was found that BAG2 was involved in a number of cellular processes, including metabolic processes, biological regulation, cellular component organization or biogenesis, immune system processes, reproductive processes, biological adhesion, rhythm process, etc. (Figure 3C).

Moreover, the transcript differences between HGC27/NC and HGC27/*shBAG2* (KD) were analyzed. A total of 196 DEPs were identified, and compared with NC group, 97 proteins were up-regulated, while 95 proteins were down-regulated (Figure 3B). Among them, the DEPs involved in the occurrence and development of cancer are detailed in the protein cluster diagram, including MAPK14, MAPK1 and CCAR2 (Figure 3D).

### BAG2 Regulates the Proliferation of Gastric Cancer Cells Through AKT/mTOR and ERK Pathway and Affects the Migration Through EMT Process

**Figure 4:**
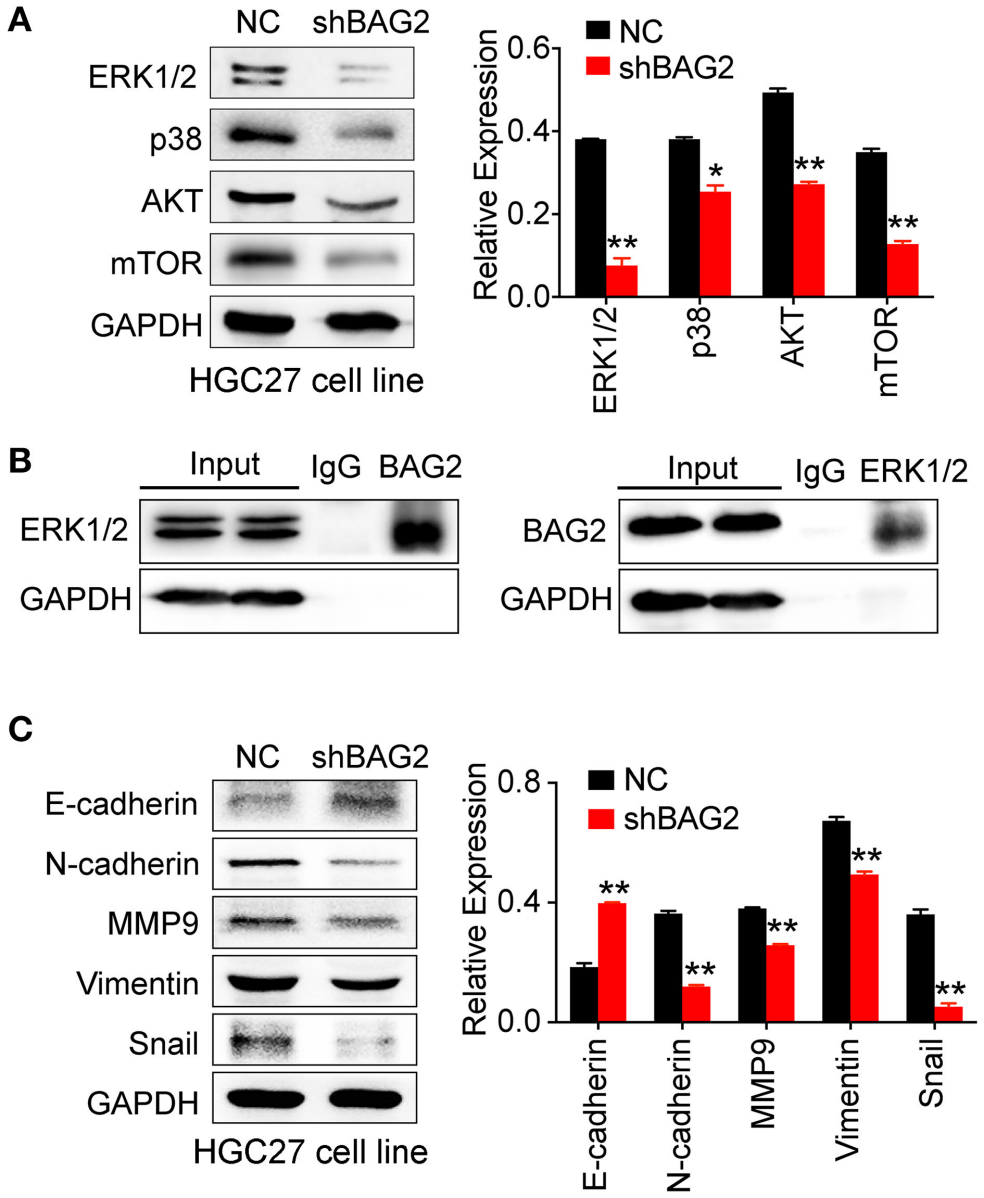
Effects of *BAG2* knockdown on AKT/mTOR pathway, ERK pathway, and EMT process. **(A)** *BAG2* knockdown significantly inhibits the expression of AKT, mTOR, p38, ERK1/2; **(B)** Immunoprecipitation result showed that BAG2 could bind to ERK1/2; **(C)** *BAG2* knockdown significantly up-regulated E-cadherin and inhibited the expression of N-cadherin, MMP9, Vimentin and Snail, which were related to EMT process. *P < 0.05; **P < 0.01.

The results of DEPs showed that BAG2 was regulated by mitogen-activated protein kinase (MAPK) and other signaling pathways, and previous cell functional experiments showed that *BAG2* knockdown could attenuate the proliferation of gastric cancer cells. Therefore, we further detected the changes of MAPK signaling pathway after knockdown of *BAG2*. We found that the expression of p38 and p42/44 (ERK1/2) in MAPK signaling pathway were significantly down-regulated, and AKT, mTOR were also notably down-regulated (Figure 4A). In addition, immunoprecipitation suggested that BAG2 could bind to ERK1/2, thereby promoting the progression of gastric cancer (Figure 4B).

Our data showed that BAG2 enhanced cell invasion and migration, which may be related to epithelial-mesenchymal transformation (EMT). Therefore, we detected the expression of E-cadherin, N-cadherin, Vimentin, MMP9, and Snail in EMT in the absence of BAG2, and found out that the expression of other EMT-related proteins decreased in varying degrees except E-cadherin (Figure 4C).

### Overexpression of miR186 Can Partially Inhibit the Expression of BAG2

**Figure 5:**
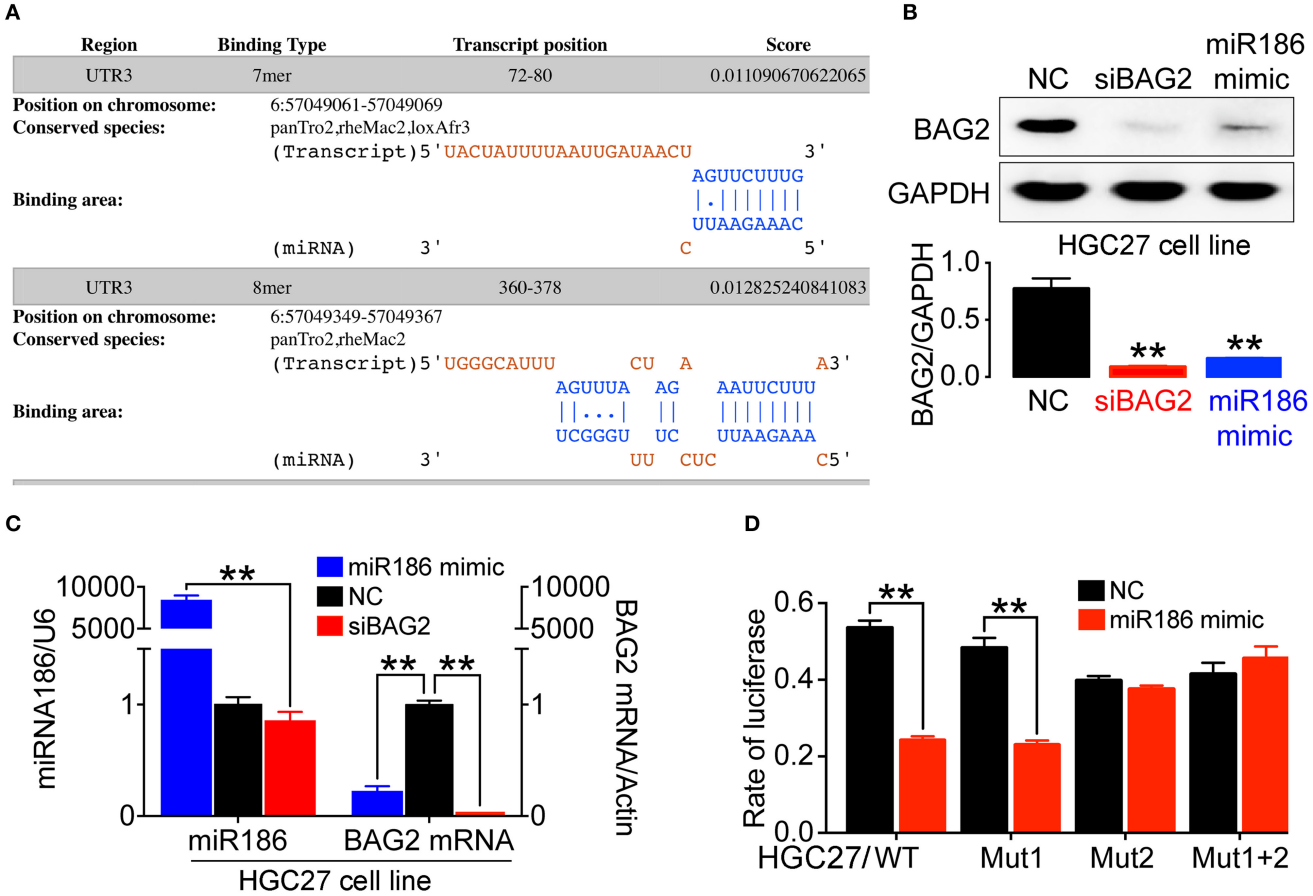
The expression of *BAG2* was regulated by miR186. **(A)** Prediction of two high score binding sites between miR186 and *BAG2*-3′UTR on DIANA website; **(B)** Overexpression of miR186 inhibits expression of BAG2 protein; **(C)** Overexpression of miR186 inhibited *BAG2* mRNA; **(D)** The effect of miR186 on the activity of luciferase reporter containing *BAG2-*3′UTR-WT*, BAG2*-3′UTR-Mut1*, BAG2*-3′UTR-Mut2 or *BAG2*-3′UTR-Mut1+2 was tested by luciferase reporter gene (**P < 0.01).

Protein expression is typically regulated by miRNAs. To analyze specific binding between miRNA sequence and *BAG2-*3′UTR, we searched several miRNA target prediction related databases, including starBase v2.0 (http://starbase.sysu.edu.cn/), miRanda (http://www.microrna.org/), PicTar (http://pictar.mdc-berlin.de/) and TargetScant (http://www.targetscan.org/), we finally determined that miR186 might specifically bind to *BAG2-*3′UTR. Moreover, in DIANA website, where multiple interaction sites between miR186 and *BAG2* may be found, we determined the first two sites for further verification (Binding sequence showed in Figure 5A).

Therefore, by using Western blot and quantitative reverse transcription polymerase chain reaction (RT-qPCR), we verified that overexpression of miR186 (miR186 mimic) can inhibit the expression of BAG2 by degrading *BAG2* mRNA (Figures 5B,C). In addition, through the Dual-luciferase Reporter Assay, we showed that miR186 could bind to *BAG2-*3′UTR through the second binding site (Figure 5D). Taken together, our data indicated that *BAG2* is a direct target gene of miR186.

### Clinical Significance of BAG2 in Gastric Cancer

**Figure 6:**
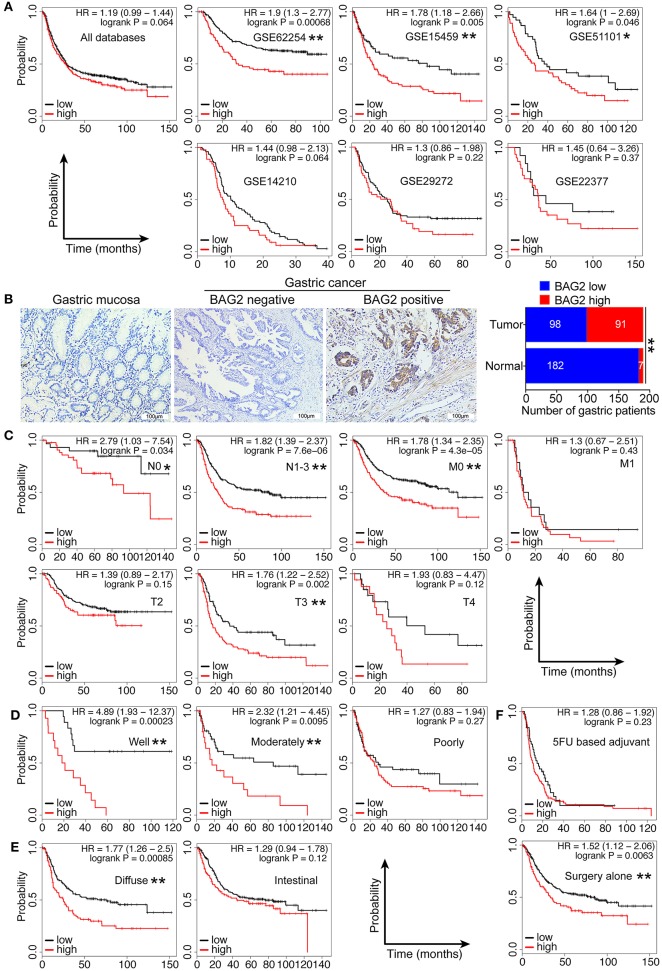
Clinical significance of high expression of BAG2 in gastric carcinoma. **(A)** Gastric cancer databases from KM-plotter shows high expression of *BAG2* mRNA in gastric cancer suggests poor prognosis; **(B)** Typical immunohistochemical staining of BAG2 in para-cancerous and cancer tissue of patients with gastric cancer (400× magnification); **(C–F)** Prognostic analysis of *BAG2* mRNA in different subgroups of gastric cancer database from KM-plotter, including T, N, M stage, differentiation degree, pathological type and treatment (*P < 0.05; ** *P* < 0.01).

In order to further confirm the clinical significance of BAG2 in gastric cancer, we specifically analyzed the prognostic significance and biological behavior of *BAG2* in gastric cancer through KM-plotter database, in which the results showed that high expression of *BAG2* indicated poor prognosis of gastric cancer (All, *P* = 0.064; GSE62254, *P* < 0.01; GSE15459, *P* < 0.01; GSE51101, *P* < 0.05; GSE14210, *P* = 0.064; GSE29272, *P* = 0.22 and GSE22377, *P* = 0.37) (Figure 6A). To verify the results of the above-mentioned bioinformatics analysis, we obtained the specimens of cancer tissue and para-cancerous gastric mucosa from 189 patients with gastric cancer. Immunohistochemistry was undertaken to confirm the localization of BAG2 protein, and it showed BAG2 mainly existed in cytoplasm (Figure 6B). Statistical results revealed that the BAG2 was low expressed in 96.3% (182/189) of normal gastric mucosa and high expressed in 48.1% (91/189) of cancer tissue (*P* < 0.01) (Figure 6B). Statistical analysis showed that the expression of BAG2 protein was significantly correlated with T stage and differentiation degree (*P* < 0.001). But there was no significant correlation between gender, N stage, M stage and clinical stages (Table 1).

**Table 1:** Clinicalpathological implications of BAG2 expression in gastric patients.

	***n* (%)**	**BAG2 expression**		***P-*value**
		**High (%)**	**Low (%)**	
Gender				0.48
Male	141 (74.6)	70 (49.6)	71 (50.4)	
Female	48 (25.4)	21 (43.8)	27 (56.3)	
T stage				0.002
T1	4 (2.1)	1 (25.0)	3 (75.0)	
T2	27 (14.3)	12 (44.4)	15 (55.6)	
T3	27 (14.3)	22 (81.5)	5 (18.5)	
T4	131 (69.3)	56 (42.7)	75 (57.3)	
N status				0.501
N0	64 (31.2)	33 (56.2)	31 (43.8)	
N1–3	125 (68.8)	58 (32.3)	67 (67.7)	
M stage				0.554
M0	146 (77.2)	72 (49.3)	74 (50.7)	
M1	43 (22.8)	19 (44.2)	24 (55.8)	
Clinical stage				0.24
I	17 (9.0)	9 (52.9)	8 (47.1)	
II	24 (12.7)	16 (66.7)	8 (33.3)	
III	105 (55.6)	47 (44.8)	58 (55.2)	
IV	43 (22.8)	19 (44.2)	24 (55.8)	
Differentiation				0.002
Well	19 (10.1)	11 (57.9)	8 (42.1)	
Moderately	45 (23.8)	31 (68.9)	14 (31.1)	
Poorly	125 (66.1)	49 (39.2)	76 (60.8)	

At the same time, we employed KM-Plotter database to analyze the correlation between the high/low expression of *BAG2* and the clinicopathological characteristics of patients with gastric cancer (Table 2). The results showed that high expression of *BAG2* in female patients was significantly higher than that in male patients (65.5 vs. 23.6%, *P* < 0.01), and that in high grade T stage (T3+T4) was remarkably higher than that in low grade (T2). In N stage and M stage, the expression of *BAG2* in high grade was higher than that in low grade (*P* < 0.01). In addition, we found that high expression of *BAG2* was positively correlated with the degree of differentiation and the expression of *HER2* (*P* < 0.01), while there was no significant difference in *BAG2* expression among different pathological types (*P* = 0.813). Furthermore, the high expression of *BAG2* in patients treated with 5FU chemotherapy was markedly higher than that in patients treated with surgery alone (74.5 vs. 27.0%, *P* < 0.01) (Table 2).

**Table 2:** Relationship between *BAG2* expression and clinicalpathological features of gastric patients from KM-plotter.

	***n* (%)**	***BAG2*** **expression**		***P-*value**
		**High (%)**	**Low (%)**	
Gender				<0.001
Male	1286 (78.8)	304 (23.6)	982 (76.4)	
Female	345 (21.2)	226 (65.5)	119 (34.5)	
T stage				<0.001
T2	400 (42.1)	111 (27.8)	289(72.3)	
T3	474 (49.9)	256 (54.0)	218 (46.0)	
T4	76 (8.0)	30 (39.5)	46 (60.5)	
N status				<0.001
N0	281 (31.2)	158 (56.2)	123 (43.8)	
N1–3	620 (68.8)	200 (32.3)	420 (67.7)	
M stage				<0.001
M0	704 (89.7)	226 (32.1)	478 (67.9)	
M1	81 (10.3)	56 (69.1)	25 (30.9)	
Clinical stage				<0.001
I	243 (16.4)	127 (52.3)	116 (47.7)	
II	251 (16.9)	68 (27.1)	183 (72.9)	
III	734 (49.5)	208 (28.3)	526 (71.7)	
IV	254 (17.1)	78 (30.7)	176 (69.3)	
Differentiation				<0.001
Well	86 (13.9)	24 (27.9)	62 (72.1)	
Moderately	173 (28.0)	43 (24.9)	130 (75.1)	
Poorly	359 (58.1)	219 (61.0)	140 (39.0)	
Pathology type				0.813
Intestinal	887 (71.6)	297 (33.5)	590 (66.5)	
Diffuse	351 (28.4)	120 (34.2)	231 (65.8)	
HER2^a^ status				<0.001
Negative	1404 (75.6)	394 (28.1)	1010 (71.9)	
Positive	453 (24.4)	315 (69.5)	138 (30.5)	
Treatment				<0.001
Surgery alone	571 (71.2)	154 (27.0)	417 (73.0)	
5-FU^b^ based adjuvant	231 (28.8)	172 (74.5)	59 (25.5)	

a *HER2, human epidermal growth factor receptor 2*.

b *5-FU, 5-Fluorouracil*.

In view of the fact that the high expression of *BAG2* associates with poor prognosis in patients with gastric cancer and that is related to several clinicopathological characteristics, we further analyzed the prognosis of high/low expression of *BAG2* in different clinicopathological subgroups. The findings showed that high expression of *BAG2* showed different degrees of poor prognosis in different T3 stage, N0 and N1-3 stage, M0 stage with statistically significance (Figure 6C). In addition, compared with poorly differentiated carcinoma, in well-differentiated and moderately differentiated carcinoma, the high expression of *BAG2* showed a significantly poor prognosis (Figure 6D). Meanwell, compared with intestinal type, the high expressed *BAG2* indicated a significantly poor prognosis in the diffuse type (Figure 6E). Finally, in the 5FU chemotherapy group, the expression of *BAG2* had no difference on the prognosis, but in the surgery alone group, the high expression of *BAG2* was significantly correlated with the poor prognosis, which could better reflect the correlation between the expression of *BAG2* and the prognosis of gastric cancer (Figure 6F).

## Discussion

Numerous evidences have shown that BAG2 plays a substantial role in the pathogenesis of neurodegenerative diseases and neurotoxicity, such as Alzheimer's disease (14), Parkinson's disease (15), sevoflurane-induced neurotoxicity (16), etc. In recent years, the role of BAG2 in cancer has significantly attracted scholars' attention. High-throughput sequencing or proteomics of different types of tumors reflects the high expression of BAG2 in cancer, including ovarian cancer (17), papillary thyroid carcinoma (18), fibrosarcoma (19) and multiple myeloma (20). Yang et al. (12) found that the overexpression of BAG2 in triple-negative breast cancer was closely associated with poor clinical results, and the unique role of *BAG2* was confirmed as an enzyme of cancer-promoting or anticancer genes. However, a limited number of studies have concentrated on the expression of BAG2 in gastric cancer.

In this study, we found that the expression of *BAG2* in gastric cancer was significantly higher than that in normal gastric mucosa according to Oncomine and Human Protein Atlas databases. Simultaneously, we preliminarily confirmed the high expression of BAG2 in gastric cancer cell lines and its low expression in gastric mucosa cell line (GES1), which laid a foundation for further study. Previous studies have shown that BAG2 mediates important cellular responses to stress, including cell cycle arrest and apoptosis (15, 21). In our study of the biological function of BAG2 in gastric cancer cells, we found an increase in apoptotic cells, the proliferation and migration of gastric cancer cells were significantly inhibited after *BAG2* knockdown. Therefore, it is important to further investigate the molecular mechanism of BAG2, playing these biological functions in gastric cancer.

Phosphatidylinositol-3-kinase/AKT/mTOR signaling pathway is considered as the main regulator of tumor (22). Abnormal mTOR signals can be observed in different types of cancer. Once overactivated, mTOR signaling promotes cell proliferation and metabolism, thereby promoting tumorigenesis and development (23). Our data confirm that BAG2 can regulate the proliferation of gastric cancer cells by activating AKT/mTOR pathway. P38 cascades are activated by various stresses or cytokines, and there are a variety of signaling pathways and a wide range of branches downstream of p38. Ueda et al. (24) found that BAG2 is directly phosphorylated by MAPKAP kinase 2 *in vitro* and *in vivo*, which is the main substrate of p38 and mediates several p38 dependent processes. Our results of protein spectrum and immunoblotting showed that *BAG2* knockdown resulted in decreased levels of p38 and ERK1/2 proteins. In addition, immunoprecipitation suggests that BAG2 can bind ERK1/2, and then promote the progression of gastric cancer.

MiRNA is a class of non-coding single-stranded RNA molecules, playing a pivotal regulatory role in the process of biological development (25). MiRNAs regulate the expression of target gene at the post-transcriptional level, mainly though paired with the base of the 3′UTR of the target mRNA in a complete or incomplete complementary manner (26). Thus, miRNAs play a vital role in tumorigenesis, biological development, organogenesis, apparent regulation, and metabolism (27, 28). Previous studies have shown that miR186 targeted *IGF-1R* in glioma (29), and *Yin Yang 1 (YY1)* and *cyclin dependent kinase 6 (CDK6)* in prostate cancer (30). In addition, *PTTG1* (31), *P2X7* (32), *FOXO1* (33), *AKAP12* (34, 35) and *Caspase-10* (36) have also been identified as direct targets of miR186 in different types of cancer in recent years. Here, we verified that overexpression of miR186 can inhibit the expression of *BAG2*. Through the dual-luciferase reporter assay, we found that miR186 directly interact with the second site (see Figure 5D) of *BAG2*-3′UTR.

EMT is a key process in tumor progression and metastasis (37). Li et al. (38) showed that overexpression of miR186 inhibits metastasis and EMT of colorectal cancer cells. Zhao et al. (39) found that miR186 strongly inhibits cell movement and EMT by down-regulating Twist1 in prostate cancer cells. Our data showed that *BAG2* knockdown might suppress intercellular EMT process by down-regulating the expression of N-cadherin, MMP9, Vimentin and Snail, thereby reducing invasion and migration of gastric cancer cells. This is consistent with the previous results of miR186 overexpression inhibiting EMT process of cancer cells (38). In conclusion, it is suggested that the process of BAG2 promoting EMT in gastric cancer cells may be partially regulated by miR186.

According to our data about EdU and apoptosis assays, the cell cycle of HGC27 was blocked, the cell proliferation decreased, and the number of apoptotic cells increased after *BAG2* knockdown. In addition, it has been reported that miR186 could inhibit cell cycle by targeting *cyclin D1, CDK2*, and *CDK6* (40), and our results support the inhibition of BAG2 expression by miR186. At the same time, in DEPs detected by iTRAQ technology, we found that cell cycle and apoptosis regulatory protein 2 (CCAR2) decreased after *BAG2* knockdown, suggesting that CCAR2 may act as a downstream factor of BAG2 to form miR186-BAG2-CCAR2 axis to regulate gastric cancer cell cycle and apoptosis. However, these findings need to be further verified.

## Conclusion

We demonstrated that BAG2 is a more valuable prognostic indicator of gastric cancer. The miR186-BAG2-ERK1/2 axis is involved in gastric cancer progression, which may provide a molecular basis for understanding of the pathophysiological function of BAG2, in addition to support the fact that these molecules could comprise a potential target for gastric cancer.

## Data Availability Statement

The datasets analyzed during the current study are available from the corresponding author on reasonable request.

## Ethics Statement

The studies involving human participants were reviewed and approved by the Ethical Committee of the First Hospital of China Medical University. The patients/participants provided their written informed consent to participate in this study.

## Author Contributions

LS, GC, CL, and KL contributed conception and design of the study. LS and GC performed the experiments. AS, ZW, and HH organized the database. ZG and WL performed the statistical analysis. LS and GC wrote the first draft of the manuscript. CL and KL wrote sections of the manuscript. All authors contributed to manuscript revision, read and approved the submitted version.

### Conflict of Interest

The authors declare that the research was conducted in the absence of any commercial or financial relationships that could be construed as a potential conflict of interest.
